# XIAP-targeting drugs re-sensitize *PIK3CA*-mutated colorectal cancer cells for death receptor-induced apoptosis

**DOI:** 10.1038/cddis.2014.534

**Published:** 2014-12-11

**Authors:** M Ehrenschwender, S Bittner, K Seibold, H Wajant

**Affiliations:** 1 Institute of Clinical Microbiology and Hygiene, University Hospital of Regensburg, Regensburg, Germany; 2 Division of Molecular Internal Medicine, Department of Internal Medicine II, University Hospital Würzburg, Würzburg, Germany

## Abstract

Mutations in the oncogenic *PIK3CA* gene are found in 10–20% of colorectal cancers (CRCs) and are associated with poor prognosis. Tumor necrosis factor-related apoptosis-inducing ligand (TRAIL) and agonistic TRAIL death receptor antibodies emerged as promising anti-neoplastic therapeutics, but to date failed to prove their capability in the clinical setting as especially primary tumors exhibit high rates of TRAIL resistance. In our study, we investigated the molecular mechanisms underlying TRAIL resistance in CRC cells with a mutant *PIK3CA* (*PIK3CA*-mut) gene. We show that inhibition of the constitutively active phosphatidylinositol-3 kinase (PI3K)/Akt signaling pathway only partially overcame TRAIL resistance in *PIK3CA*-mut-protected HCT116 cells, although synergistic effects of TRAIL plus PI3K, Akt or cyclin-dependent kinase (CDK) inhibitors could be noted. In sharp contrast, TRAIL triggered full-blown cell death induction in HCT116 *PIK3CA*-mut cells treated with proteasome inhibitors such as bortezomib and MG132. At the molecular level, resistance of HCT116 *PIK3CA*-mut cells against TRAIL was reflected by impaired caspase-3 activation and we provide evidence for a crucial involvement of the E3-ligase X-linked inhibitor of apoptosis protein (XIAP) therein. Drugs interfering with the activity and/or the expression of XIAP, such as the second mitochondria-derived activator of caspase mimetic BV6 and mithramycin-A, completely restored TRAIL sensitivity in *PIK3CA*-mut-protected HCT116 cells independent of a functional mitochondrial cell death pathway. Importantly, proteasome inhibitors and XIAP-targeting agents also sensitized other CRC cell lines with mutated *PIK3CA* for TRAIL-induced cell death. Together, our data suggest that proteasome- or XIAP-targeting drugs offer a novel therapeutic approach to overcome TRAIL resistance in *PIK3CA*-mutated CRC.

Colorectal cancer (CRC) is among the three most common malignancies worldwide.^1^ Pathophysiologically, CRC development been linked to the acquisition of oncogenic mutations such as alterations in the phosphoinositide-3 kinase (PI3K)/Akt pathway. PI3K converts phosphatidylinositol 4,5-bisphosphate to phosphatidylinositol 3,4,5-trisphosphate, thereby generating a docking site for pleckstrin homology domain containing proteins such as Akt/PKB. In CRC, approximately 10–20% of tumors exhibit mutations in the p110*α* catalytic subunit (predominantly H1047R and E545K substitutions in the *PIK3CA* gene), causing constitutive PI3K/Akt activation^2^ and worsening clinical outcome.^3^

Tumor necrosis factor-related apoptosis-inducing ligand (TRAIL) emerged as a promising anti-cancer agent, capable of selectively inducing cell death in tumor cells.^4^ TRAIL binding to TRAIL receptor 1 (TRAIL-R1) or TRAIL-R2 induces formation of a chain-like death-inducing signaling complex (DISC). This allows stepwise caspase-8 activation and initiates a cascade of proteolytic cleavage events finally activating caspase-3 and triggering the execution phase of apoptosis.

In so-called type I cells, initial caspase-8-mediated cleavage of caspase-3 efficiently triggers further autocatalytic caspase-3 processing to the mature heterotetrameric p12-p17 molecule. In type II cells, however, X-linked inhibitor of apoptosis protein (XIAP) inhibits processing of the caspase-3 p19 intermediate to the p17 subunit of the mature enzyme. Death receptor-induced apoptosis in these cells therefore relies on a mitochondria-dependent amplification loop that is triggered by caspase-8-mediated cleavage of the BH3-interacting domain death agonist (Bid) to tBid.^5^ tBid activates Bcl2-associated X protein (Bax) and Bcl2-antagonist/killer (Bak), enabling pore-formation in the outer mitochondrial membrane and release of apoptogenic factors such as cytochrome *c* and second mitochondria-derived activator of caspase (SMAC).^6^ The pro-apoptotic effect is at least twofold: cytochrome *c* associates with apoptotic protease-activating factor 1 (Apaf-1), forming a molecular scaffold for caspase-9 activation (‘apoptosome'), which in turn boosts downstream effector caspase activation. Synergistically, SMAC neutralizes cytosolic inhibitors of apoptosis proteins (IAPs), such as cIAP1, cIAP2 and especially XIAP.^7^

High levels of IAPs or deregulated expression of Bcl2 family proteins are common in human cancers and often confer apoptosis resistance. This hampers efficacy of TRAIL-based therapies and to date, the therapeutic benefit of TRAIL in clinical trials is indeed rather limited.^8^

We have recently found that mutant *PIK3CA* licensed TRAIL and CD95L to induce an amoeboid morphology in CRC cells, which is associated with increased invasiveness *in vitro.*^9^ Here, we show that targeting of the aberrantly active PI3K/Akt signaling pathway in TRAIL resistant, *PIK3CA*-mutated CRC cells only partially restored death receptor-triggered apoptosis induction. We identified impaired caspase-3 maturation by XIAP as the underlying molecular mechanism of TRAIL resistance in HCT116 *PIK3CA*-mut cells. Inhibition of XIAP or the proteasome efficiently restored TRAIL sensitivity irrespective of mitochondria-dependent death signal amplification. Together, our results indicate that targeting XIAP or the proteasome in CRC with PIK3CA mutations may offer a promising strategy to exploit the therapeutic potential of TRAIL in cancer therapy.

## Results

### Mutant *PIK3CA* shifts TRAIL and Fc-CD95L signaling from apoptosis induction to pro-survival signaling

**Figure 1:**
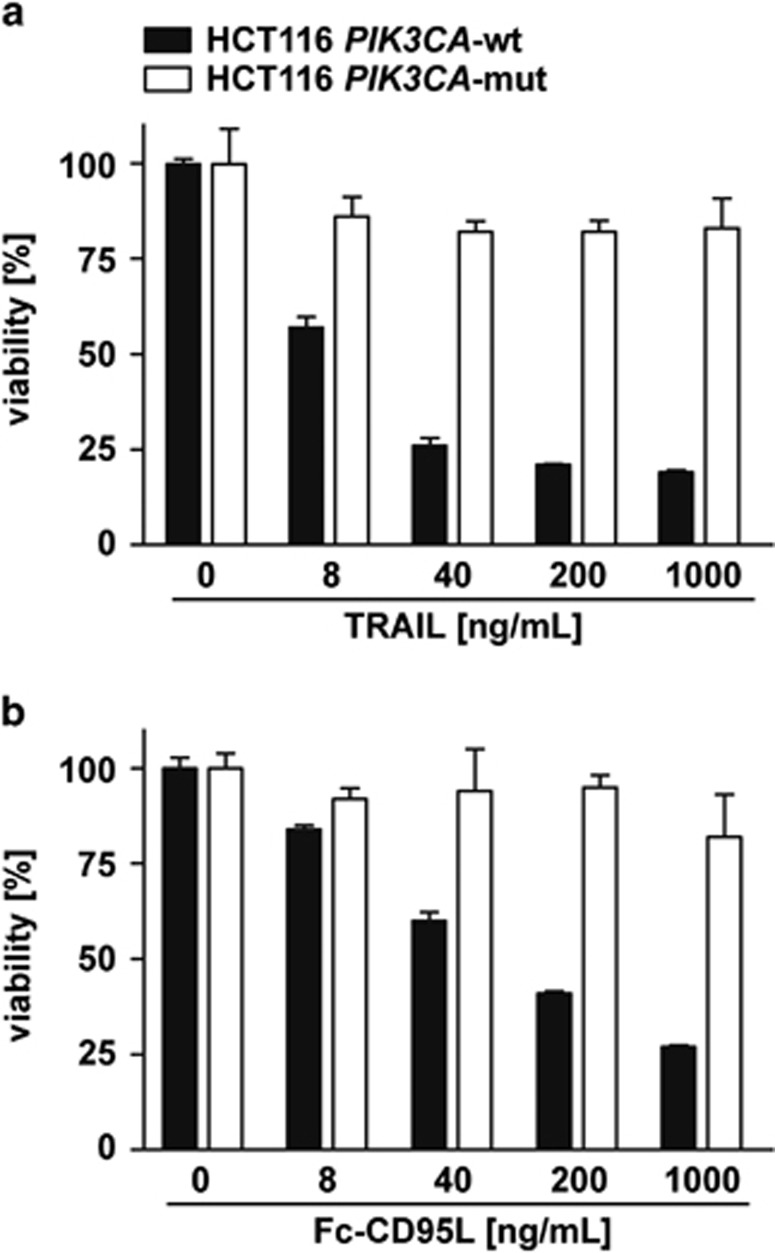
*PIK3CA*-mut renders HCT116 cells resistant to TRAIL- and Fc-CD95L-induced cell death. HCT116 *PIK3CA*-wt and HCT116 *PIK3CA*-mut cells were seeded in 96-well plates and challenged the next day in triplicates with the indicated concentrations of (**a**) TRAIL and (**b**) Fc-CD95L. Cell viability was determined by 3-[4,5-dimethylthiazol-2-yl]-2,5-diphenyl tetrazolium bromide staining 18 h after stimulation. Results are given as mean values±S.D. from three experiments

Gene targeting of *PIK3CA* in the CRC cell line HCT116 revealed that exclusive expression of a PIK3CA allele harboring an activating H1047R substitution (HCT116 *PIK3CA*-mut) in exon 20 conferred resistance to TRAIL-induced apoptosis (Figure 1a).^9, 10^ In sharp contrast, the isogenic cell line with a wild-type PIK3CA allele (HCT116 *PIK3CA*-wt) died in a dose-dependent manner after TRAIL stimulation (Figure 1a; ED50-value ~10 ng/mL). Importantly, Samuels *et al.* reported TRAIL resistance in two PIK3CA mutant clones,^10^ thereby ruling out simple clone-to-clone variations. *PIK3CA*-mut-mediated protection was not limited to TRAIL, but also observable upon treatment with Fc-CD95L (Figure 1b). This suggested that the *PIK3CA*-mut-granted resistance is not restricted to a single death receptor/ligand pair, but acts as a more general protective mechanism against death receptor-induced apoptosis.

**Figure 2:**
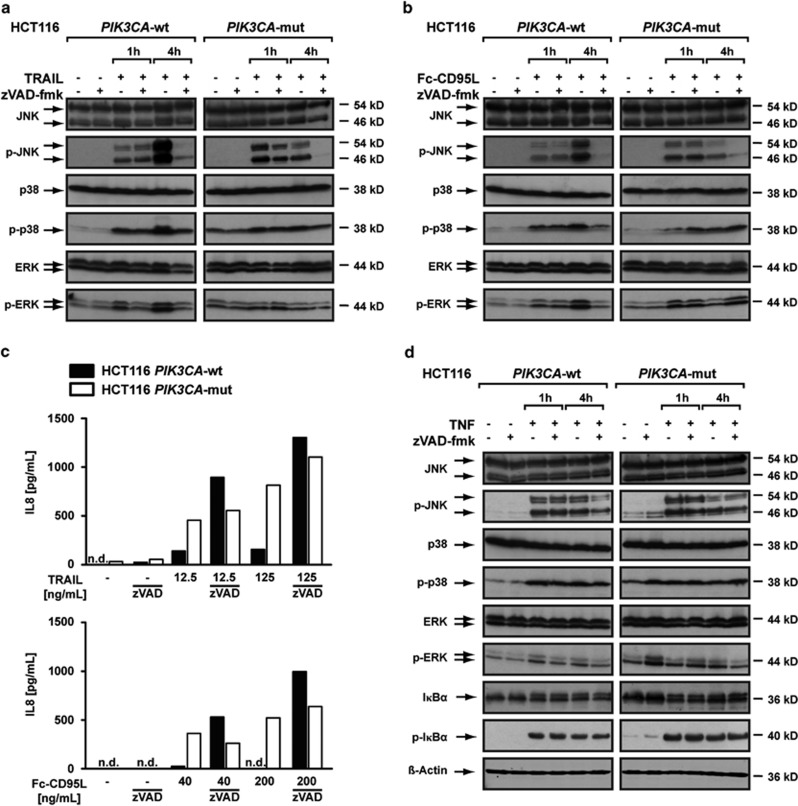
TRAIL and Fc-CD95L elicit a pro-inflammatory response in *PIK3CA*-mut-protected cells. (**a**, **b** and **d**) HCT116 *PIK3CA*-wt and HCT116 *PIK3CA*-mut cells were stimulated with (**a**) TRAIL (50 ng/mL), (**b**) Fc-CD95L (200 ng/mL) or (**d**) TNF (100 ng/mL) for the indicated periods of time in the presence and absence of the pan-caspase inhibitor zVAD-fmk (100 *μ*M, added 1 h before stimulation). Subsequently, cells were washed, lysed and analyzed by western blotting using antibodies specific for the indicated proteins. Detection of *β*-actin served as a loading control. Lysates from HCT116 *PIK3CA*-wt and HCT116 *PIK3CA*-mut cells have been run on the same gel for each condition. (**c**) HCT116 *PIK3CA*-wt and HCT *PIK3CA*-mut cells were seeded in triplicates in 96-well plates and challenged the next day with the indicated concentrations of TRAIL or Fc-CD95L for 6 h in the presence and absence of zVAD-fmk (100 *μ*M, added 1 h before stimulation). Supernatants were harvested and IL-8 levels were measured using ELISA. Results given as mean and representative for two experiments performed. n.d., not detectable

Beside cell death induction, TRAIL-R1/-R2 and CD95 also strongly activate pro-inflammatory and pro-survival pathways, especially when cell death is blocked.^11, 12^ We therefore investigated pro-inflammatory TRAIL- and Fc-CD95L-triggered responses in HCT116 *PIK3CA*-wt and HCT116 *PIK3CA*-mut cells. In principle, no differences regarding activation of the mitogen-activated protein kinases, JNK, p38 and ERK in both cell lines (Figures 2a and b) were observed. Caspase inhibition using zVAD-fmk barely affected early but almost completely abolished sustained TRAIL-induced JNK activation. This presumably reflects previously reported differences in the caspase dependency of the early and late phase of death receptor-induced biphasic JNK activation.^13, 14^ Consistently, TRAIL- and Fc-CD95L-induced caspase activation in apoptosis-susceptible HCT116 *PIK3CA*-wt cells was accompanied by high phospho-JNK levels.

We observed robust NF-*κ*B activation and induction of the NF-*κ*B target gene IL-8 in HCT116 *PIK3CA*-mut cells (Figure 2c). In HCT116 *PIK3CA*-wt cells, TRAIL or Fc-CD95L challenge predominately induced apoptosis and IL-8 production was critically dependent on blocking caspase activation with zVAD-fmk (Figure 2c). Importantly, the impaired NF-*κ*B response in HCT116 *PIK3CA*-wt cells upon TRAIL or Fc-CD95L treatment did neither reflect gross abnormalities in the NF-*κ*B signaling pathway *per se*, nor cell line-specific (HCT116 *PIK3CA*-wt *versus PIK3CA*-mut) differences in TRAIL receptor- or CD95-associated signaling pathways for the following reasons: treatment of HCT116 *PIK3CA-*wt and the *PIK3CA-*mut counterparts with the primarily pro-inflammatory ligand TNF exhibited comparable levels of phospho-I*κ*B*α*, thereby ruling out general defects in the activation of the NF-*κ*B pathway (Figure 2d). Additionally, induction of IL-8 in zVAD-fmk-protected HCT116 *PIK3CA*-wt cells after TRAIL or Fc-CD95L treatment was at least as efficient as in HCT116 *PIK3CA*-mut cells (Figure 2c).

Together, our findings demonstrated that TRAIL and Fc-CD95L induced a pro-inflammatory response in apoptosis-resistant HCT116 *PIK3CA*-mut cells.

### Pharmacological inhibition of Akt, PI3K and CDK only partially sensitizes *PIK3CA*-mut-protected cells to TRAIL

**Figure 3:**
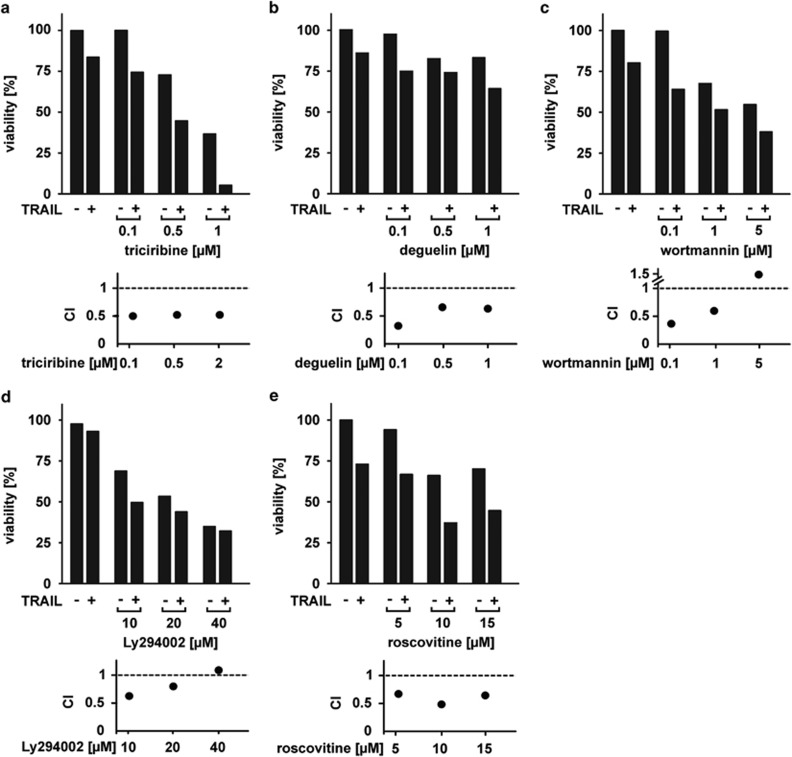
Pharmacological targeting of PI3K, Akt and CDKs only partially sensitizes HCT116 *PIK3CA*-mut cells toward TRAIL-induced cell death. HCT116 *PIK3CA*-mut cells were seeded in triplicates in 96-well plates and stimulated the next day with 125 ng/mL TRAIL in the presence and absence of (**a**) triciribine, (**b**) deguelin, (**c**) wortmannin, (**d**) Ly294002 or (**e**) roscovitine. Inhibitors were added 1 h before TRAIL stimulation. After 18 h, cell viability was determined by 3-[4,5-dimethylthiazol-2-yl]-2,5-diphenyl tetrazolium bromide staining. For each combination of TRAIL plus inhibitor, the corresponding combination index (CI) value was calculated. CI-values >1 indicate a synergistic effect (see also Materials and Methods section).^13^ Results for the viability assays are given as mean and representative for two experiments performed. For calculation of CI-values, means derived from the viability assays were used

We next investigated mechanisms to overcome TRAIL resistance in HCT116 *PIK3CA*-mut cells. As previous work demonstrated constitutive activation of the PI3K/Akt signaling axis in HCT116 *PIK3CA*-mut cells,^10, 15^ we evaluated the contribution of this pathway to TRAIL resistance. Pre-treatment of HCT116 *PIK3CA*-mut cells with the Akt inhibitors triciribine (Figure 3a) or deguelin (Figure 3b) resulted only in a partial re-sensitization to TRAIL-induced cell death. Additionally, increasing concentrations of triciribine and deguelin exhibited cytotoxic effects themselves. This potentially hampers recognition of true synergistic effects of TRAIL plus Akt inhibitor treatment. We therefore quantitatively assessed synergism by calculating a combination index (CI) for all used TRAIL plus inhibitor combinations. Briefly, CI-values >1 indicate a synergistic effect of TRAIL in combination with a pharmacological inhibitor.^13^ Decreasing CI-values correlate with enhanced synergism of the two compounds. CI-values above 1 are indicative for antagonism.

In terms of cell death induction, synergistic effects of TRAIL combined with the Akt inhibitors triciribine (CI_0.1 *μ*M triciribine_=0.49, CI_0.5 *μ*M triciribine_=0.51, CI_2 *μ*M triciribine_=0.52) and deguelin (CI-values of 0.34, 0.65 and 0.63 for 0.1 *μ*M, 0.5 *μ*M and 1 *μ*M concentrations of deguelin, respectively) were observed (Figures 3a and b, lower panel).

Similar observations were made when challenging HCT116 *PIK3CA*-mut cells with TRAIL in the presence of PI3K-inhibiting molecules. Wortmannin plus TRAIL exhibited synergistic CI-values (Figure 3c, lower panel) for wortmannin concentrations up to 1 *μ*M (CI_0.1 *μ*M wortmannin_=0.35, CI_1 *μ*M wortmannin_=0.59), but this synergistic effect was lost at higher wortmannin concentrations (CI_5 *μ*M wortmannin_=1.55). Ly294002, another PI3K inhibitor, also acted synergistically with TRAIL in concentrations up to 20 *μ*M (CI_10 *μ*M Ly294002_=0.53, CI_20 *μ*M Ly294002_=0.81). Again, increased cytotoxic effects by Ly294002 itself were reflected by higher CI-values (CI_40 *μ*M Ly294002_=1.09) and correlated with observations from the corresponding viability assays (Figure 3d). A recent report demonstrated that TRAIL resistance could be overcome by inhibition of CDK 9.^8^ Combining TRAIL with the CDK-interfering agent roscovitine (Figure 3e) revealed synergistic CI-values (CI_5 *μ*M roscovitine_=0.67, CI_10 *μ*M roscovitine_=0.48, CI_15 *μ*M roscovitine_=0.64).

Importantly, although we were able to clearly demonstrate synergistic effects of combinatorial treatments of TRAIL and an Akt, PI3K or CDK inhibitor, the re-sensitization to TRAIL-induced cell death was only partial. As robust cell death induction in transformed cells is crucial for effective TRAIL-based anti-cancer therapy, we concluded that interfering with the PI3K/Akt signaling axis or CDKs is most probably insufficient to fully exploit the therapeutic potential of TRAIL in PIK3CA-mutant CRC cells.

### Proteasome inhibition fully restores TRAIL-mediated cell death induction in HCT 116 *PIK3CA*-mut cells

**Figure 4:**
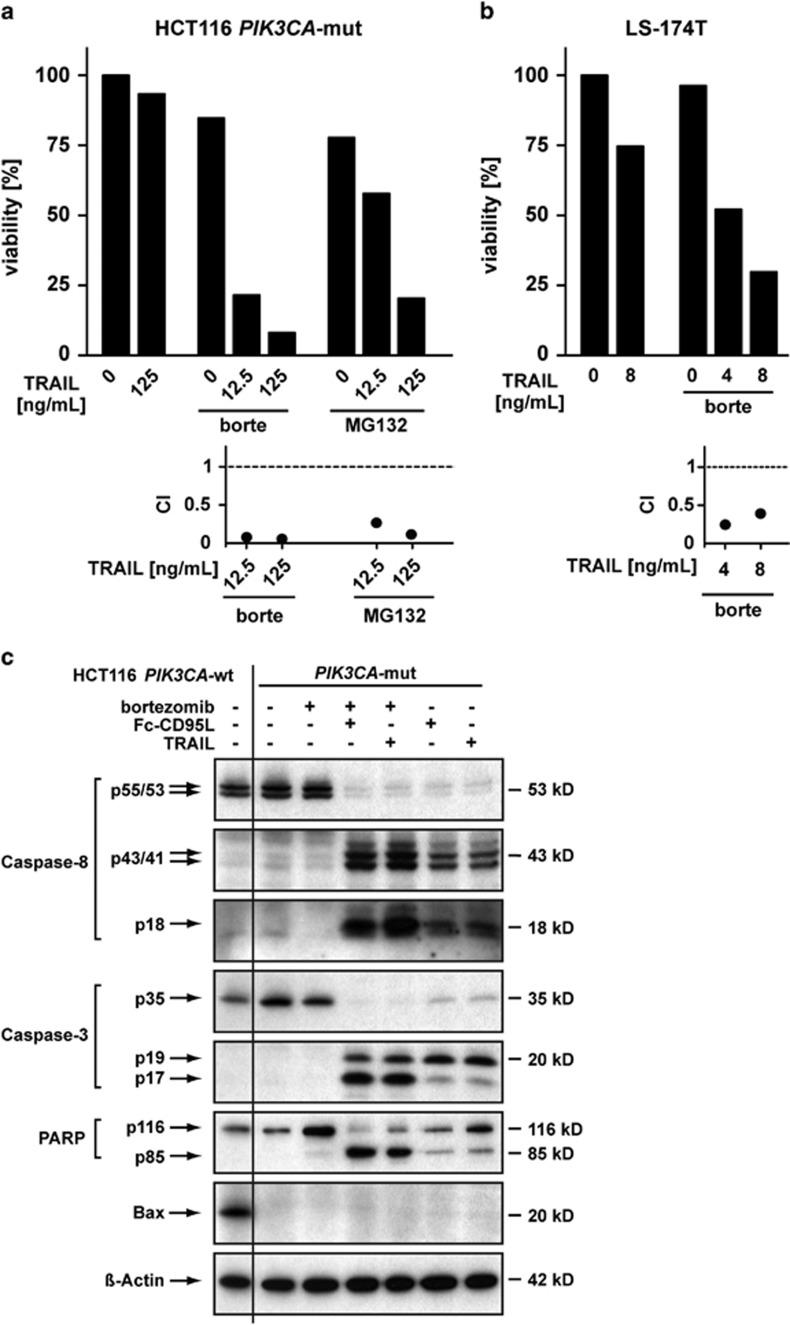
Proteasome inhibition restores TRAIL susceptibility in HCT 116 *PIK3CA-*mut cells and allows full-blown caspase-3 processing. (**a**) HCT116 *PIK3CA*-mut cells were seeded in triplicates in 96-well plates and challenged the next day with 12.5 or 125 ng/mL TRAIL in the presence and absence of 10 nM bortezomib (borte) or 100 nM MG132. Proteasome inhibitors were added 1 h before TRAIL treatment. Cell viability was determined by 3-[4,5-dimethylthiazol-2-yl]-2,5-diphenyl tetrazolium bromide (MTT) staining after 18 h. For each combination of TRAIL plus proteasome inhibitor, the corresponding CI-value was calculated. Results for the viability assays are given as mean and representative for two experiments performed. For calculation of CI-values, means derived from the viability assays were used. (**b**) LS174-T cells were seeded in triplicates in 96-well plates and challenged the next day with 4 or 8 ng/mL TRAIL in the presence and absence of 80 nM borte. After 18 h, cell viability was determined by MTT staining and the corresponding CI-values were calculated. Results for the viability assays are given as mean and representative for two experiments performed. For calculation of CI-values, means derived from the viability assays were used. (**c**) HCT116 *PIK3CA*-mut cells were stimulated with TRAIL (125 ng/mL) or Fc-CD95L (200 ng/mL) overnight in the presence and absence of borte (10 nM, added 1 h before stimulation). Cells were harvested, washed and subsequently analyzed by western blotting using antibodies specific for the indicated proteins. Detection of *β*-actin served as a loading control. HCT116 *PIK3CA*-wt cells (first lane) were included to demonstrate cell line-specific differences in Bax expression between *PIK3CA*-mut and *PIK3CA*-wt cells

Inhibition of the proteasome has been found to break apoptosis resistance in a variety of tumor entities including CRC.^14^ We therefore investigated whether blockade of the ubiquitin-proteasome system affects TRAIL-induced cell death in HCT116 *PIK3CA*-mut cells. Indeed, pre-treatment with the proteasome inhibitors bortezomib or MG132 resulted in almost full-blown cell death induction (Figure 4a, upper panel), whereas the proteasome inhibitors alone showed only modest cytotoxic activity. Accordingly, the CI-values (Figure 4a, lower panel) indicated a very strong synergism for TRAIL in combination with bortezomib (CI_12.5 ng/mL TRAIL_=0.01, CI_125 ng/mL TRAIL_=0.01) and MG132 (CI_12.5 ng/mL TRAIL_=0.14, CI_125 ng/mL TRAIL_=0.25). Importantly, sensitization for TRAIL-induced cell death by proteasome inhibition was not restricted to HCT116 *PIK3CA*-mut cells and was also evident in the PIK3CA-mutant CRC cell line LS-174T (H1047R substitution, CI-values from 0.21 to 0.48; Figure 4b).

Caspases are the predominant executors of TRAIL-induced cell death and we therefore analyzed caspase activation in HCT116 *PIK3CA*-mut cells following TRAIL challenge. Whereas caspase-8 was completely processed, finally yielding the active p18 fragment (Figure 4c), processing of caspase-3 stopped at the inactive p19 intermediate. Treatment of HCT116 *PIK3CA*-mut cells with Fc-CD95L revealed similar results, thereby arguing against a TRAIL-R1/-R2-restricted phenomenon. These findings are in accordance with our previous work that localized blockade of the apoptotic cascade in HCT116 *PIK3CA*-mut cells downstream of DISC formation and caspase-8 activation.^9^ Interestingly, pre-treatment of *PIK3CA*-mut-protected cells with bortezomib licensed TRAIL or Fc-CD95L to complete processing of caspase-3 with concomitant cleavage of PARP, an indicator for ongoing apoptosis. Of note, sensitization toward TRAIL-induced cell death did not depend on the pro-apoptotic protein Bax, as Bax expression in HCT116 *PIK3CA*-mut cells was virtually absent compared with HCT116 *PIK3CA*-wt cells and did not change upon proteasome inhibition (Figure 4c). Reduced Bax expression levels in CRC cells harboring activating PIK3CA-mutations have been described earlier,^15^ but the relevance for cell death induction when the ubiquitin-proteasome system is blocked remained obscure.

Taken together, our data provide evidence that impaired caspase-3 processing is causative for hindering TRAIL- or Fc-CD95L-induced cell death in HCT116 *PIK3CA*-mut cells. Proteasome inhibition efficiently overcomes the blockade in caspase-3 activation and enables robust, Bax-independent cell death induction.

### TRAIL stimulation reduces cellular XIAP levels in bortezomib-treated *PIK3CA*-mut-protected cells

**Figure 5:**
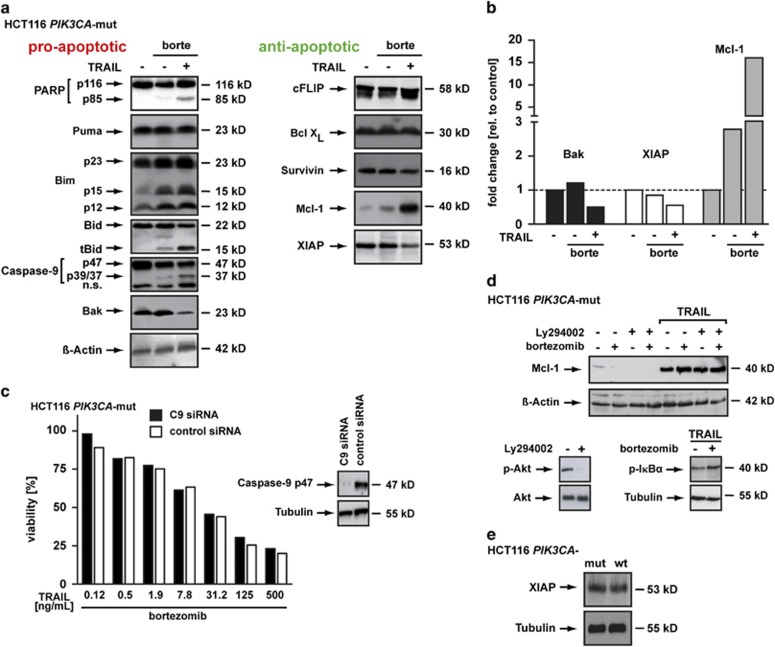
TRAIL lowers XIAP levels and potently induces cell death in proteasome-blocked HCT116 *PIK3CA*-mut cells. (**a**) HCT116 *PIK3CA*-mut cells were challenged with 125 ng/mL TRAIL for 6 h in the presence and absence of 10 nM bortezomib (borte) or left untreated. Cells were harvested, lysed and subsequently analyzed using western blotting with specific antibodies for the indicated proteins. (**b**) Decrease in Bak and XIAP levels as well as increase in Mcl-1 levels were quantified using densitometry. (**c**) HCT116 *PIK3CA*-mut cells were seeded in six-well plates and transfected with caspase-9-specific or non-targeting siRNA oligonucleotides the next day. 24 h after transfection, cells were transferred into 96-well plates and another 24 h later challenged in triplicates with different concentrations of TRAIL. Knockdown efficacy was controlled by western blotting using specific antibodies 48 h after siRNA transfection. Results given as mean and representative for two experiments performed. (**d**) Upper panel: HCT116 *PIK3CA*-mut cells were challenged with 125 ng/mL TRAIL in the presence and absence of the PI3K inhibitor Ly294002 (10 *μ*M) and the proteasome inhibitor borte (10 nM). Subsequently, cells were harvested, lysed and analyzed for Mcl-1 or β-actin using specific antibodies. Detection of β-actin served as a loading control. Lower panel: HCT116 *PIK3CA*-mut cells were treated with PI3K inhibitor Ly294002 (2 h, 10 *μ*M) or the proteasome inhibitor borte (10 nM). Borte-treated cells were subsequently challenged with TRAIL (50 ng/mL) for 2 h. Cells were harvested and subjected to western blot analysis using antibodies with specificity for the indicated proteins. (**e**) XIAP levels in lysates from HCT116 *PIK3CA*-mut and HCT116 *PIK3CA*-wt cells were analyzed using western blotting with specific antibodies for the indicated proteins. Detection of tubulin served as a loading control

The sensitizing effect of proteasome inhibitors to cell death in various malignant tumors has been linked to changes in cellular levels of pro- or anti-apoptotic proteins. In CRC, the underlying molecular mechanism is poorly understood. Therefore, we analyzed cellular levels of pro- and anti-apoptotic proteins in HCT116 *PIK3CA*-mut cells upon TRAIL treatment in the presence and absence of bortezomib (Figure 5a, left panel). We did not observe accumulation of apoptosis-promoting BH3-only proteins such as Puma and Bcl2-interacting mediator of cell death (Bim), but detected TRAIL-induced cleavage of Bid into tBid, which was even more pronounced with bortezomib. Concomitantly, cleavage of the pro-form of caspase-9 was observable, indicating involvement of the intrinsic, mitochondria-dependent cell death pathway. This was astonishing, because HCT116 *PIK3CA*-mut cells are virtually Bax deficient (Figure 4c).^15^ Therefore, formation of a pore complex releasing cytochrome *c* for caspase-9 activation via the apoptosome should be hampered. We also analyzed the expression level of Bak, an alternative channel-forming protein in the outer mitochondria membrane. Interestingly, Bak levels upon bortezomib and TRAIL treatment decreased by ~50% (Figure 5b), arguing against a critical role of the Bax/Bak system in the bortezomib-mediated sensitization of *PIK3CA*-mut-protected HCT116 cells to TRAIL. However, as a previous report found that TRAIL-induced cell death under proteasome inhibition crucially depended on a functional intrinsic apoptotic pathway,^16^ we assessed the role of the mitochondria-dependent cell death pathway for TRAIL susceptibility in bortezomib-treated HCT116 *PIK3CA*-mut cells.

Apoptosome-mediated caspase-9 activation is central for intrinsic cell death induction. We therefore hypothesized that knockdown of caspase-9 should rescue bortezomib-treated HCT116 *PIK3CA*-mut cells from TRAIL-induced cell death. To our surprise, knockdown of caspase-9 failed to do so (Figure 5c), indicating that in our experimental setting caspase-9 and Bid cleavage represented rather an epiphenomenon of massively ongoing caspase-mediated proteolysis but not a driving force for apoptosis induction. In fact, cleavage of caspase-9 is not only unnecessary for its activation but it is also insufficient to produce an active enzyme.^17^

To that point, elevations in apoptosis-promoting molecules seemed unlikely to be the molecular basis for bortezomib-induced TRAIL sensitization. Consequently, we analyzed changes in the levels of anti-apoptotic proteins in HCT116 *PIK3CA*-mut cells upon treatment with TRAIL plus bortezomib (Figure 5a, right panel). Although bortezomib is generally capable to decrease levels of anti-apoptotic proteins such as cFLIP,^18^ no significant changes in the levels of cFLIP, long splice variant of Bcl-X (Bcl-X_L_) and Survivin were detectable in our cellular system (Figure 5a). Of note, bortezomib increased myeloid cell leukemia 1 (Mcl-1) expression approximately 2.7-fold and approximately 15-fold in combination with TRAIL (Figure 5b). This result was unexpected as the strong upregulation of an anti-apoptotic protein obviously failed to rescue bortezomib-treated HCT116 *PIK3CA*-mut cells from TRAIL-induced cell death. Enhanced expression of Mcl-1 in HCT116 *PIK3CA-*mut cells was solely dependent on TRAIL. Interfering with PI3K/Akt pathway using Ly294002 or bortezomib-mediated blockade of the proteasome had no negative effect on Mcl-1 expression, although shutdown efficiency of the targeted pathways was confirmed (Figure 5d, lower panel) by decrease of phospho-Akt (Ly294002) and accumulation of phospho-I*κ*B*α* following TRAIL stimulation (bortezomib).

Beside changes in Mcl-1 levels, TRAIL challenge of bortezomib-treated HCT116 *PIK3CA*-mut cells also reduced cellular XIAP levels (Figure 5a, right panel) by ~50% (Figure 5b). Notably, cellular XIAP levels showed no significant differences between HCT116 *PIK3CA*-wt and *PIK3CA*-mut cells (Figure 5e).

In sum, bortezomib alone did not significantly change the levels of the pro- and anti-apoptotic proteins we surveyed. Intriguingly, TRAIL challenge of proteasome-blocked HCT116 *PIK3CA*-mut cells reduced XIAP levels and was accompanied by complete caspase-3 activation and apoptosis induction (Figure 4). XIAP has been implicated in the regulation of caspase-3 processing, essentially allowing cleavage of pro-caspase-3 to the p19 intermediate but blocking further processing to the fully active fragment p17.^19^ As this strikingly resembled blocked caspase-3 activation in HCT116 *PIK3CA*-mut cells, we hypothesized that XIAP holds a crucial role in TRAIL resistance of *PIK3CA*-mut-protected cells.

### Drugs interfering with expression and activity of XIAP re-sensitize mutant *PIK3CA* CRC cells to TRAIL-induced cell death

**Figure 6:**
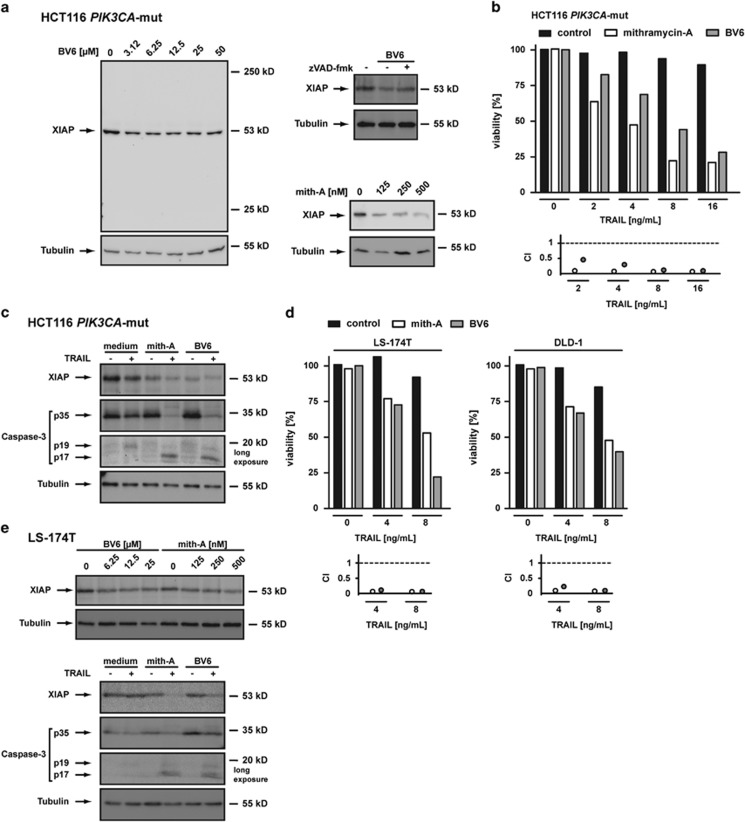
XIAP is essential for TRAIL resistance in HCT116 *PIK3CA*-mut cells. (**a**) HCT116 *PIK3CA*-mut cells were treated with the indicated concentrations of BV6 (left panel) or mithramycin-A (mith-A; lower right panel) for 18 h. To block caspase activation in BV6-treated cells (upper right panel), zVAD-fmk (100 *μ*M) was added 1 h before BV6 treatment. Subsequently, cells were harvested, lysed and analyzed by western blotting for the indicated proteins using specific antibodies. Detection of tubulin served as loading control. (**b**) HCT116 *PIK3CA*-mut cells were seeded in 96-well plates and challenged the next day with the indicated concentrations of TRAIL in the presence and absence of mith-A (500 nM, added 1 h before stimulation) or BV6 (12.5 *μ*M, added 1 h before stimulation). 18 h after TRAIL challenge, cell viability was determined by 3-[4,5-dimethylthiazol-2-yl]-2,5-diphenyl tetrazolium bromide (MTT) staining. Results are given as mean and representative for two experiments, for calculation of CI-values, means derived from the viability assays were used. (**c**) HCT116 *PIK3CA*-mut cells were treated with mith-A (500 nM, added 1 h before stimulation) or BV6 (12.5 *μ*M, added 1 h before stimulation) and subsequently challenged with 8 ng/mL TRAIL for 6 h. Cells were harvested, lysed and subsequently analyzed using western blotting with specific antibodies for the indicated proteins. Detection of tubulin served as a loading control. (**d**) LS174-T and DLD-1 cells were seeded in 96-well plates and challenged the next day with the indicated concentrations of TRAIL in the presence and absence of mith-A (500 nM, added 1 h before stimulation) or BV6 (25 *μ*M, added 1 h before stimulation). 18 h after TRAIL challenge, cell viability was determined by MTT staining. Results are given as mean and representative for two experiments, for calculation of CI-values, means derived from the viability assays were used. (**e**) Upper panel: LS-174T cells were treated with the indicated concentrations of mith-A or BV6 for 18 h. Subsequently, cells were harvested, lysed and analyzed by western blotting for the indicated proteins using specific antibodies. Detection of tubulin served as loading control. Lower panel: LS-174T cells were treated with mith-A (500 nM, added 1 h before stimulation) or BV6 (25 *μ*M, added 1 h before stimulation) and subsequently challenged with 8 ng/mL TRAIL for 6 h. Cells were harvested, lysed and subsequently analyzed using western blotting with specific antibodies for the indicated proteins. Detection of tubulin served as a loading control

Next, we asked if lowering XIAP expression/activity with molecules such as mithramycin-A (mith-A)^20^ or the SMAC-mimetic BV6^21^ sensitizes HCT116 *PIK3CA*-mut cells to TRAIL. BV6 and mith-A reduced XIAP levels in HCT116 *PIK3CA*-mut cells in a dose-dependent manner (Figure 6a). For BV6, XIAP reduction has been attributed to caspase-mediated cleavage during ongoing apoptosis.^22^ However, the mode of action potentially differs in HCT116 *PIK3CA*-mut cells, as BV6-reduced XIAP levels in the presence of the pan-caspase inhibitor zVAD-fmk (Figure 6a, right panel) and no XIAP cleavage products were detectable (Figure 6a).

Lowering XIAP levels significantly sensitized *PIK3CA*-mut-protected HCT116 cells to TRAIL-induced cell death (Figure 6b). Of note, the ED50-value was approximately 4 ng/mL for TRAIL plus mithramycin and approximately 8 ng/mL for TRAIL plus BV6, almost perfectly matching TRAIL-susceptible HCT116 *PIK3CA*-wt cells (Figure 1a).^9^ In accordance with the significantly enhanced TRAIL-induced loss of viability of mithramycin or BV6 pre-treated *PIK3CA*-mut-protected cells, the CI-values for combinatorial treatment indicated strong synergistic effects, ranging from 0.01 to 0.03 for TRAIL plus mithramycin and from 0.46 to 0.11 for TRAIL plus BV6 (Figure 6b, lower panel). TRAIL-induced cell death was accompanied by complete caspase-3 processing upon mith-A or BV6 pre-treatment (Figure 6c). Notably, TRAIL concentrations as low as 8 ng/mL efficiently activated caspase-3. Our findings were not restricted to HCT116 *PIK3CA*-mut cells. Mith-A and BV6 also robustly sensitized LS-174T and DLD-1 to TRAIL-induced cell death with CI-values ranging from 0.002 to 0.08 (Figure 6d). Likewise, BV6 and mith-A reduced XIAP levels in LS-174T (Figure 6e) and DLD-1 cells (data not shown) in a dose-dependent manner and allowed complete TRAIL-induced caspase-3 activation in both cell lines (Figure 6e and data not shown).

## Discussion

PIK3CA-mutations in CRC limit therapeutic options and are associated with poor prognosis.^3^ Isogenic HCT116 cell lines expressing solely a wild-type or mutant (H1047R) PIK3CA allele exhibited a constitutively activated PI3K/Akt-pathway, high-level resistance against TRAIL-induced cell death^10^ and increased invasiveness *in vitro* and *in vivo*.^9, 15^ TRAIL resistance was essentially attributed to constitutive PI3K activation and reduced Bax expression.^15, 23^ We confirmed that mutant *PIK3CA* shifts TRAIL and Fc-CD95L signaling from cell death induction to pro-survival signaling via robust NF-*κ*B activation. The latter was poor in HCT116 *PIK3CA*-wt cells (Figure 2c) and most likely attributable to efficient shut down of the classical NF*κ*B pathway through caspase-mediated cleavage of signaling intermediates.^24^ In line with this, rescuing TRAIL or Fc-CD95L-treated *PIK3CA*-wt cells with the pan-caspase inhibitor zVAD-fmk allowed at least as efficient NF-*κ*B activation in HCT116 *PIK3CA*-wt compared with HCT116 *PIK3CA*-mut cells (Figure 2c). Our findings have a fundamental impact on therapeutic approaches toward mutant *PIK3CA*-bearing CRC cells, as TRAIL-induced pro-inflammatory signaling potentially fosters tumor formation and has been associated with increased invasiveness *in vitro.*^9^ Consequently, TRAIL-based therapies in this tumor entity may even be contraindicated unless TRAIL resistance can be efficiently overcome.

To date, attempts to break TRAIL resistance included among others blockade of NF-*κ*B signaling, proteasome inhibition and inhibition of histone deacetylases.^25, 26, 27^ In HCT116 *PIK3CA*-mut cells, interfering with the activated PI3K/Akt pathway significantly reduced cell survival under growth factor deprivation stress.^15^ We therefore analyzed the effect of PI3K, Akt and CDK inhibitors combined with TRAIL and demonstrated a synergistic mode of action regarding cell death induction (Figure 3). The observed synergism is potentially a TRAIL-specific phenomenon, as in an earlier study, treatment of mutant *PIK3CA* CRC cells with PI3K inhibitors and cytotoxic drugs such as doxorubicin failed to synergistically increase cell death induction, although proliferation ceased.^28^ However, re-sensitization of HCT116 *PIK3CA*-mut cells to TRAIL with any of these inhibitors was not full-blown but only partial. Potentially, nonspecific or ineffective pharmacological inhibition could be causative for inefficient sensitization but seemed unlikely, as multiple inhibitors targeting the PI3K/Akt signaling axis used at various concentrations revealed comparable results.

In any case, incomplete re-sensitization leaves the possibility that TRAIL-based therapies might trigger tumorigenic effects in the surviving population. In order to find a more efficient method to sensitize *PIK3CA*-mut-protected cells to TRAIL, we examined the influence of proteasome inhibition in combination with TRAIL treatment (Figure 4a). Cell viability was barely affected by the proteasome inhibitors bortezomib or MG132 alone. In sharp contrast, addition of TRAIL resulted in nearly complete cell death induction, which was more pronounced in the presence of bortezomib compared with MG132. Importantly, bortezomib-mediated sensitization for TRAIL-induced cell death was not restricted to HCT116 *PIK3CA*-mut cells but also occurred in the PIK3CA-mutant CRC cell lines LS-174T and DLD-1.

Mechanistically, several models have been proposed to explain TRAIL sensitization after proteasome-blockade, such as (a) downregulation of the anti-apoptotic protein cFLIP with subsequently enhanced activation of caspase-8;^18^ (b) stabilization of the pro-apoptotic proteins Bax^29^ or tBid^16^ and (c) increased levels of the pro-apoptotic BH3-only proteins Bik and Bim.^30^ However, none of these mechanisms was applicable to the bortezomib-induced TRAIL sensitivity in HCT116 *PIK3CA*-mut cells, as in the presence and absence of bortezomib and/or TRAIL (a) cFLIP levels (Figure 5a) as well as (b) Bax levels (Figure 4c) remained constant; tBid generation and caspase-9 cleavage were dispensable for cell death induction (Figure 5c) and (c) Bim levels (Figure 5a) did not change significantly (Bik was not detectable, data not shown). Admittedly, a wide-scale proteomic analysis of bortezomib-induced changes in the expression of pro- and anti-apoptotic proteins might reveal additional candidates.

Surprisingly, despite robust TRAIL-induced cell death induction in bortezomib-treated HCT116 *PIK3CA*-mut cells, the anti-apoptotic protein Mcl-1 was significantly upregulated (Figures 5a and b). Although bortezomib alone only modestly increased Mcl-1 levels, TRAIL treatment was capable to significantly enhance Mcl-1 expression independent of PI3K/Akt or proteasome inhibition (Figure 5d). Accumulation of Mcl-1 upon proteasome inhibition is in line with a previous study, demonstrating an increase of this protein in various malignant tumor cell lines (including CRC) upon MG132 treatment.^31^ Because of the accumulation of Mcl-1, proteasome inhibition might not under all circumstances have anti-tumoral activity. In fact, increased Mcl-1 levels in proteasome-blocked CRC^31^ and neuroblastoma cells^32^ elicited pro-tumoral or tumor-protective effects. Mechanistically, Mcl-1 can bind to and sequester Bak, thereby interfering with Bax/Bak-mediated pore formation in the outer mitochondrial membrane and inhibiting the mitochondrial cell death pathway.^33^ Even massive TRAIL-induced upregulation of Mcl-1 in HCT116 *PIK3CA*-mut cells failed to prevent cell death in the presence of bortezomib. This pointed to a mitochondria-independent mode of bortezomib-mediated TRAIL sensitization, which is in line with our finding that downregulation of caspase-9 did not protect bortezomib-treated HCT116 *PIK3CA*-mut cells from TRAIL-induced cell death. In sum, our results argued for a bortezomib-granted but mitochondria-independent mode of TRAIL-induced cell death.

Interestingly, HCT116 cells are considered to be type II cells. According to our findings, however, TRAIL-induced cell death in bortezomib-treated HCT116 *PIK3CA*-mut cells resembled type I cells. Earlier reports demonstrated that in the absence of TRAIL bortezomib-induced cell death depends on Bax. Bax-deficient HCT116 cells are consequently bortezomib resistant.^34^ In contrast, a recent study elegantly showed that proteasome inhibitors effectively overcame TRAIL resistance even in Bax- and Bax-/Bak-deficient HCT116 cells with a dysfunctional mitochondrial cell death pathway.^35^ These data suggested that bortezomib switched the mode of cell death from type II to type I. This resembled our observations in *PIK3CA*-mut-protected HCT116 cells, as bortezomib fully re-sensitized to TRAIL-induced cell death independent of the intrinsic cell death pathway. But in contrast to the cited study,^35^ HCT116 *PIK3CA*-mut cells are genetically neither Bax- nor Bak-deficient.

Blocked caspase-3 activation upon TRAIL stimulation at the level of the inactive p19 intermediate (Figure 4c) pointed to a potential role of XIAP in death resistance of HCT116 *PIK3CA*-mut cells. The p19 fragment but not the active p17 fragment of caspase-3 is negatively regulated by XIAP.^36^ XIAP contains three caspase-interacting BIR (baculovirus IAP repeat) domains and is itself a substrate of caspase-8. Caspase-8-generated cleavage fragments of XIAP inhibit further processing of caspase-3 intermediates^36^ by competitive binding of BIR1 and BIR2 (BIR1-2) to a neo-epitope in the caspase-3 p19 fragment.^19^ This inhibition can be surmounted by robust caspase-3 activation and cleavage of XIAP in a manner that functionally inactivates the BIR1-2 fragment.^36^ In TRAIL-resistant melanoma cells, for example, caspase-3-mediated XIAP cleavage acts as a positive feedback loop that sensitizes to TRAIL-induced cell death.^37^ In line with this, TRAIL-induced caspase-3 activation in bortezomib, BV6 and mith-A pre-treated HCT116 *PIK3CA*-mut cells further enhanced the initially observed caspase-independent reduction of XIAP of these drugs (Figures 5a and 6c). This presumably reflects proteolytic cleavage of the caspase-3 substrate XIAP.

In addition to competitive caspase inhibition, the E3-ligase activity of XIAP inhibits caspase-3 activation by ubiquitination of the active p17 fragment with subsequent proteasomal degradation.^38^ TRAIL stimulation of proteasome-blocked HCT116 *PIK3CA*-mut cells resulted in the appearance of the active caspase-3 p17 fragment (Figure 4c), indicating ongoing proteasomal degradation in cells with intact ubiquitin-proteasome system. TRAIL resistance in HCT116 *PIK3CA*-mut cells might therefore be regulated through the E3-ligase activity of XIAP.

Accordingly, decreasing cellular XIAP levels in HCT116 *PIK3CA*-mut, LS-174T and DLD-1 cells using the SMAC-mimetic BV6^21^ potently enhanced TRAIL sensitivity (Figures 6b and d) and was accompanied by complete TRAIL-induced caspase-3 activation (Figures 6c and e). BV6 not only inhibits XIAP but also induces autoubiquitination and proteasomal degradation of cIAP1 and cIAP2,^22^ opening the possibility that degradation of cIAP1 and cIAP2 rather than XIAP inhibition is causative for the synergistic effect of BV6 and TRAIL. This cannot be fully excluded, but seems unlikely as cIAP1 and cIAP2 are only poor competitive inhibitors of caspase-3,^39^ and, in contrast to XIAP, cIAP1 targets the p19 fragment not the p17 fragment of caspase-3 for proteasomal degradation.^40^ Mith-A essentially lowers XIAP levels by blockade of Sp1-mediated transcription^20^ and thus affects XIAP activity by a different mechanism than BV6. This strengthens the idea that XIAP is the most relevant target of BV6 in the enhancement of TRAIL-induced cell death. Mith-A might possibly also affect other apoptosis regulatory proteins beside XIAP. A more direct evaluation of the relevance of XIAP using siRNA experiments technically failed, however, because of poor knockdown efficacy.

**Figure 7:**
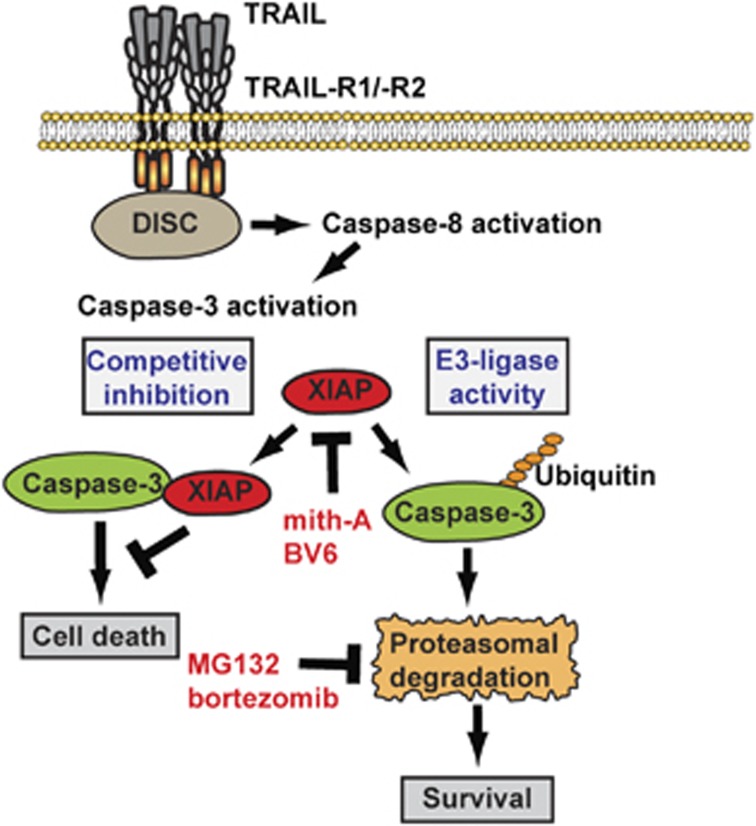
Central role of XIAP in TRAIL-resistant *PIK3CA*-mut CRC cells. In HCT116 *PIK3CA*-mut cells, XIAP critically controls TRAIL resistance by proteasomal degradation of the active p17 fragment of caspase-3. Consequently, proteasome inhibition allows complete caspase-3 activation upon TRAIL stimulation. The effect is twofold: first, robust caspase-3 activation allows execution of apoptotic cell death. Second, active caspase-3 reduces cellular XIAP levels through proteolytic cleavage in a positive feedback loop, thereby amplifying TRAIL-induced cell death. Notably, the decisive role of XIAP in this context is independent from the mitochondrial cell death pathway. DISC, death-inducing signaling complex; mith-A, mithramycin-A

Taken together, our study points to XIAP as a potential druggable molecule to overcome of TRAIL resistance in *PIK3CA*-mut-protected CRC cells (summarized in Figure 7). Our data provide experimental evidence that combinatorial treatment approaches with TRAIL- and XIAP-targeting molecules such as SMAC mimetics or XIAP-selective agents potentially extend the currently limited therapeutic options of *PIK3CA*-mutated CRC.

## Materials and Methods

### Cell lines, antibodies and reagents

LS-174T and DLD-1 cells were purchased from the German Collection of Microorganisms and Cell Culture (DSMZ, Braunschweig, Germany). HCT116 *PIK3CA*-wt and HCT116 *PIK3CA*-mut cells were a kind gift from Bert Vogelstein (Johns Hopkins University, Baltimore, MA, USA). These isogenic cell lines exclusively harbor either a wild-type (HCT116 *PIK3CA*-wt) or a mutated allele (H1047R, HCT116 *PIK3CA*-mut) for *PIK3CA*. Cells were cultured in RPMI 1640 medium (PAA, Pasching, Austria) supplemented with 10% fetal calf serum (PAA). Fc-CD95L was produced in HEK293 cells and purified by affinity chromatography targeting an internal Flag epitope. Protein G agarose beads coupled to anti-Flag mAb were purchased from Sigma (Deisenhofen, Germany). Human recombinant TRAIL was purchased from Apronex (Jesenice u Prahy, Czech Republic). Roscovitine, Ly294002 and wortmannin were purchased from Adipogen (Liestal, Switzerland), deguelin from Cayman chemicals (Ann Arbor, MI, USA) and mith-A from AppliChem (Darmstadt, Germany). BV6 (originally described in Varfolomeev *et al.*^22^) was synthesized as described elsewhere.^41^ zVAD-fmk was obtained from Bachem (Heidelberg, Germany), MG132 from Merck (Darmstadt, Germany), bortezomib from US Biological (Swampscott, MA, USA). Antibodies specific for caspase-3, caspase-8, caspase-9, JNK, phospho-JNK, ERK, phospho-ERK, p38, phospho-p38, I*κ*B*α*, phospho-I*κ*B*α*, Puma, Bim, Bid, Bak, cFLIP, Bcl-X_L_, Survivin, Mcl-1 and XIAP were purchased from Cell Signaling (Beverly, MA, USA). Anti-Bax was from Santa Cruz (Santa Cruz, CA, USA), anti-*β*-actin from Sigma, anti-tubulin from Dunn Labortechnik (Asbach, Germany). Anti-RIP1 and anti-PARP were purchased from BD Biosciences (Heidelberg, Germany).

### Cell viability assay

Cells (4 × 10^4^ per well) were seeded in 96-well plates and challenged the next day with the indicated concentrations of TRAIL or Fc-CD95L in triplicates. Cell viability was determined 18 h after stimulation using 3-[4,5-dimethylthiazol-2-yl]-2,5-diphenyl tetrazolium bromide staining.

### Western blot analysis

Phosphorylation of proteins was analyzed as follows: cells were harvested in medium, spun down, and were directly dissolved in 4x Laemmli sample buffer (8% SDS, 0.1 M dithiothreitol, 40% (v/v) glycerol, 0.2 M Tris, pH 8.0) supplemented with phosphatase inhibitor cocktails-I and -II (Sigma). Subsequently, lysates were sonicated and boiled for 5 min at 96 °C. Protein separation for western blot analyses was achieved by SDS-polyacrylamide gel electrophoresis. After transfer to nitrocellulose membranes, nonspecific binding sites were blocked by incubation in Tris-buffered saline containing 0.1% Tween 20 and 5% dry milk. Membranes were incubated with primary antibodies of the specificity of interest. Antigen–antibody complexes were visualized using secondary, horseradish peroxidase-conjugated antibodies (Dako, Hamburg, Germany) and the ECL western blotting detection system (Pierce, Rockford, IL, USA).

For preparation of Triton X-100 lysates, cells were washed and resuspended in lysis buffer (30 mM Tris–HCl, 1% (v/v) Triton X-100, 10% (v/v) glycerol, 120 mM NaCl, pH 5) supplemented with complete protease inhibitor cocktail (Roche Diagnostics GmbH, Mannheim, Germany). After incubation for 20 min on ice, lysates were cleared by centrifugation (20 min, 14 000 × *g*). After addition of Laemmli buffer, samples were boiled for 5 min at 96 °C and separated by SDS-polyacrylamide gel electrophoresis.

### siRNA transfection

Knockdown of caspase-9 was performed using siRNA oligonucleotides (Qiagen, Hilden, Germany). Briefly, cells were seeded in six-well plates (2.5 × 10^5^ cells per well) and 200 pmol of caspase-9-specific or control siRNA were transfected the next day using Lipofectamine 2000 (Invitrogen, Carlsbad, CA, USA) according to the manufacturer's instructions. 48 h post-transfection, knockdown efficacy was determined by western blot analysis.

### Calculation of CI

The CI-values were calculated using the median effect/CI isobologram method.^13^ In this model, CI-values <1 are considered to be synergistic, whereas CI-value >1 indicate antagonistic effects. Synergistic effects can be further graded based on the calculated CI-value: <0.1 very strong synergism, 0.1–0.3 strong synergism, 0.3–0.7 synergism, 0.7–0.85 moderate synergism, 0.85–0.9 slight synergism, 0.9–1.1 no synergism but nearly additive effects.^13^ All CI calculations were performed using the freely available software CompuSyn version 1.0.

### Determination of IL-8 production

For measuring IL-8 secretion, cells (2 × 10^4^ per well) were seeded in 96-well tissue culture plates and cultured overnight. Medium was changed and the indicated concentrations of TRAIL, Fc-CD95L or medium were added for 6 h in the presence and absence of 100 *μ*M zVAD-fmk. The supernatant was collected and IL-8 was quantified using an ELISA (BD Biosciences). All groups were analyzed as triplicates.

### Densitometry

Intensities of protein bands were quantified using the open source software ImageJ 1.47v (Wayne Rasband; National Institutes of Health, Bethesda, MD, USA).

## Abbreviations

Bak: Bcl2-antagonist/killer
Bax: Bcl2-associated X protein
Bcl-X_L_: long splice variant of Bcl-X
Bid: BH3-interacting domain death agonist
Bim: Bcl2-interacting mediator of cell death
CDK: cyclin-dependent kinase
CI: combination index
CRC: colorectal cancer
DISC: death-inducing signaling complex
IAP: inhibitor of apoptosis protein
Mcl-1: myeloid cell leukemia 1
mith-A: mithramycin-A
PI3K: phosphatidylinositol-3 kinase
SMAC: second mitochondria-derived activator of caspase
TRAIL: tumor necrosis factor-related apoptosis-inducing ligand
XIAP: X-linked inhibitor of apoptosis
